# Mitochondria-Associated Membranes (MAMs) are involved in Bax mitochondrial localization and cytochrome c release

**DOI:** 10.15698/mic2019.05.678

**Published:** 2019-03-15

**Authors:** Alexandre Légiot, Claire Céré, Thibaud Dupoiron, Mohamed Kaabouni, Nadine Camougrand, Stéphen Manon

**Affiliations:** 1 Institut de Biochimie et de Génétique Cellulaires, UMR 5095 CNRS & Université de Bordeaux, Campus Carreire, CS61390, 1 Rue Camille Saint-Saëns, 33077 Bordeaux, France.

**Keywords:** Bax, apoptosis, mitochondria associated membranes, mitochondria, cytochrome c, ERMES, density gradients, Saccharomyces cerevisiae

## Abstract

The distribution of the pro-apoptotic protein Bax in the outer mi-tochondrial membrane (OMM) is a central point of regulation of apoptosis. It is now widely recognized that parts of the endoplasmic reticulum (ER) are closely associated to the OMM, and are actively involved in different signaling processes. We addressed a possible role of these domains, called Mitochon-dria-Associated Membranes (MAMs) in Bax localization and function, by ex-pressing the human protein in a yeast mutant deleted of MDM34, a ERMES (ER-Mitochondria Encounter Structure) component. By affecting MAMs stabil-ity, the deletion of MDM34 altered Bax mitochondrial localization, and de-creased its capacity to release cytochrome c. Furthermore, the deletion of MDM34 decreased the size of an incompletely released, MAMs-associated pool of cytochrome c.

## INTRODUCTION

Apoptosis, the major programmed cell death pathway in animals, plays a central role during development, and along the whole life, by mediating the elimination of dispensable or potentially dangerous cells. Apoptosis is also involved in the response of cells to toxic molecules, such as anti-tumor drugs. Apoptosis alterations are involved in developmental defects, tumor progression, and the resistance to anti-cancer treatments ([1, 2] for reviews).

The permeabilization of the outer mitochondrial membrane (OMM) is a central event during the intrinsic pathway of apoptosis, that is normally activated by anti-tumor treatments. It is controlled by proteins of the Bcl-2 family, among which the pro-apoptotic protein Bax is directly involved in the permeabilization process [3]. Following an apoptotic signal, Bax is redistributed from a mostly cytosolic to a mitochondrial localization that favors permeabilization. This redistribution is associated to the exposure of hydrophobic domains [4], the dimerization and the oligomerization of the protein [5], and changes in the interactions with different partners, including anti-apoptotic proteins of the Bcl-2 family, BH3-only proteins, and other regulators such as the mitochondrial receptor Tomm22 ([6–9] for reviews).

Mitochondria-Associated Membranes (MAMs) are domains of the endoplasmic reticulum (ER) that are in close contact with the OMM [10, 11]. They have been observed through microscopy and isolated as a membrane compartment containing elements from both ER and OMM. They are involved in biogenesis processes, like the biosynthesis and transfer of phospholipids from ER to mitochondria [12], in signaling processes, like ceramide synthesis [13] and Ca^2+^-movements between the ER and mitochondria [14], or in degradation processes, like mitophagy [15]. MAMs are dynamic structures that are done and undone, depending on the physiological status of the cell ([16–18] for reviews).

We addressed a possible role of MAMs in Bax localization and redistribution in healthy cells and during apoptosis. Under certain conditions, a small proportion of Bax was found in the ER [19, 20]. The hypothesis that MAMs stability could contribute to the regulation of Bax movements between different compartments thus deserves investigations. As underlined above, a difficulty comes from the fact that MAMs are not a stable well-defined compartment but are dependent on other cellular processes such as mitochondrial dynamics, Ca^2+^-signaling, or mitophagy. In mammalian cells, it is then difficult to evaluate the contribution of MAMs in Bax localization independently from these cellular processes, that also modulate apoptosis.

The heterologous expression of Bax, and other Bcl-2 family members, in yeast is a simplified cellular model to investigate molecular mechanisms underlying the interaction of Bax with mitochondria [21]. In yeast, MAMs stability depends on the ERMES (ER-Mitochondria Encounter Structure) complex. ERMES is formed of four proteins, Mmm1p, Mdm10p, Mdm12p and Mdm34p ([17, 22] for review). A fifth protein, Gem1p, belonging to the Miro family (Mitochondria Rho GTPase) contributes to the regulation of the complex [23]. The role of ERMES in lipid transfer between ER and OMM has been largely documented ([16] for review), but remains discussed [24]. Also, the role of individual proteins remains unclear. Furthermore, the deletion of *MMM1, MDM10* or *MDM12* genes leads to a dramatically altered phenotype, including a rapid loss of mitochondrial DNA, making functional studies difficult [25,26]. However, the deletion of MDM34 leads to a marginally altered phenotype, with functional mitochondria (this study). We thus expressed Bax in a MDM34-null mutant, to evaluate the dependence of Bax localization and function on MAMs stability. Our results show that Bax mitochondrial localization is decreased when MAMs were destabilized through the deletion of MDM34. Furthermore, we observed a pool of incompletely released, MAMs-associated cytochrome c, the size of which was decreased in the Δ*mdm34* strain.

## RESULTS

The substitution P168A stimulates Bax mitochondrial localization and ability to permeabilize the OMM for cytochrome c when it is expressed in yeast [27–29] and in glioblastoma cells [30]. Also, purified recombinant Bax-P168A was more efficient than the wild-type protein to permeabilize isolated yeast or human mitochondria [31]. The difference between the two proteins was largely attenuated when assayed on liposomes [31], suggesting that additional components were needed to emphasize the distinct activities of the two proteins. Interestingly, a mutant P168G forms stable dimers not inserted into the mitochondrial membrane [32]. This substitution does not simply change the position of the hydrophobic helix α9, but has more general effects on the whole conformation of Bax [33]. Considering these data, we studied whether other factors that have not been considered until now could be involved in the ability of Bax-P168A to interact with mitochondria.

We built a W303-1B strain carrying a Δ*mdm34::kanMX4* deletion (that will be named Δ*mdm34* below). This strain did not exhibit major alterations of cell growth and viability, opposite to mutants carrying deletions of MMM1, MDM10 or MDM12 [25, 26, 34–37]. Indeed, Δ*mdm34* grew in YNB supplemented with the non-fermentable carbon source lactate, showing that mitochondria were functional. Compared to wild-type, the doubling time was marginally increased (4h15 *v/s* 4h) and the cell density at the stationary phase was slightly decreased (2x10^8^ *v/s* 2.4x10^8^ cells/mL). This suggested that the deletion of MDM34 did not impair the replication/transmission of mitochondrial DNA. Also, the mitochondrial network was distinctly formed, although slightly altered, in the Δ*mdm34* mutant (Fig. S1).

**Figure 1:**
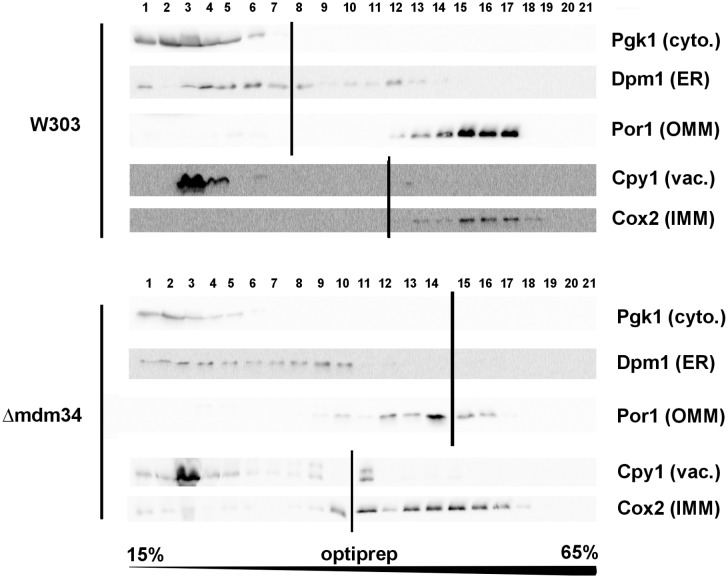
FIGURE 1: The deletion of MDM34 destabilizes MAMs. Cell extracts were separated on a 15-65% gradient of Optiprep. The gradient was resolved into 21 fractions and analyzed by western-blot for the presence of indicated markers. Vertical lines separate different gels. Data shown are from extracts obtained at the same time for each strain, and are representative of four independent experiments.

Cellular extracts of the W303-1B and Δ*mdm34* strains were first analyzed on Optiprep density gradients (**Fig. 1**). Differences between the two strains were in line with the expected phenotype of Δ*mdm34*. In wild-type extracts, a minor population of ER (visualized by the presence of Dpm1) was found at a higher density (around fraction 12) than the main ER population (fractions 3-8). This additional fraction was absent in Δ*mdm34* extracts. However, fractions containing mitochondrial proteins (Por1 and Cox2) were found at lower densities in Δ*mdm34* (fractions 10-17) than in wild-type (12-17). Consequently, the Δ*mdm34* strain still contained fractions where ER and mitochondrial proteins were both present (namely fraction 10), albeit at a lower density than in wild-type (fractions 12-13).

**Figure 2:**
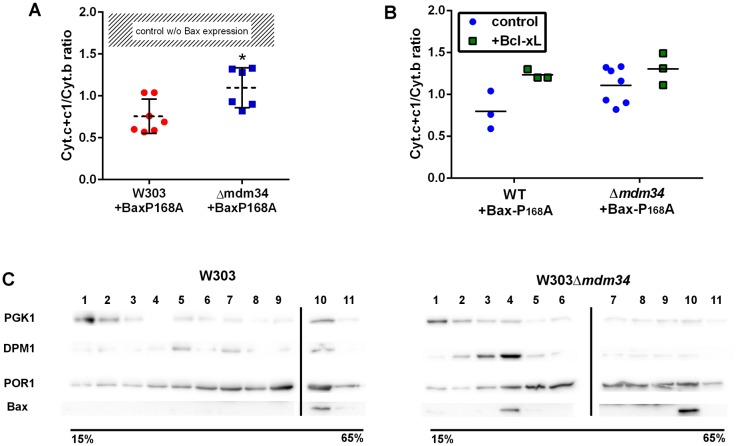
FIGURE 2: Bax-P168A is partly retained in ER, and has a decreased ability to release cytochrome c in Δ*mdm34* cells. **(A)** Cytochrome c+c1/Cytochrome b ratios measured on mitochondria isolated from wild-type or Δ*mdm34* cells expressing Bax-P168A. Each point represents a single mitochondria preparation. The hatched zone corresponds to the typical values found on mitochondria preparations from cells that do not express Bax [27–39]. *: p<0.05 (unpaired Student t-test). **(B)** Cytochrome c+c1/Cytochrome b ratios measured on mitochondria preparations isolated from strains co-expressing Bax-P168A and Bcl-xL. **(C)** Separation of whole extracts from cells expressing Bax-P168A on a 15-65% sucrose density gradient. Vertical lines mark the separation between different gels. Data are representative of four independent experiments.

To test the involvement of these modifications on Bax relocation and activity, the mutant Bax-P168A was comparatively expressed in wild-type and Δ*mdm34* yeast strains. In yeast, the activity of Bax is quantitatively measured through the amount of residual mitochondrial cytochrome c by redox spectrophotometry: less mitochondrial cytochrome c indicates a higher Bax activity. The activity of Bax-P168A was slightly but significantly decreased when expressed in the Δ*mdm34* strain (**Fig. 2A**). A possible explanation could be that the deletion of MDM34 would make mitochondria less susceptible to be permeabilized (because of, e.g., a different lipid composition). The co-expression of the anti-apoptotic protein Bcl-xL partially inhibited Bax-P168A-induced cytochrome c release (**Fig. 2B**), and abolished the difference between both strains. This showed that, when Bax was inhibited, the permeability of wild-type and Δ*mdm34* mitochondria was the same, and the differential effect of Bax-P168A was actually caused by an alteration of Bax function in Δ*mdm34* mitochondria, and not linked to a general property making them less likely to be permeabilized. Cell extracts from both strains expressing Bax-P168A were analyzed on sucrose density gradients. Although sucrose-based gradients showed more cross-contamination than Optiprep-based gradients, they provided a better discrimination between ER-containing fractions in wild-type and Δ*mdm34* strains (**Fig. 2C**). Under these conditions, a population of Bax-P168A was associated with the ER in the Δ*mdm34* strain (fraction 4) but not in the wild-type (**Fig. 2C**). This suggested that some Bax-P168A remained associated with the ER compartment, resulting in the decreased ability to release cytochrome c. However, the difference between the localization of Bax-P168A in the wild-type and Δ*mdm34* strain was modest and thus not translated into any significant change in yeast cell growth and survival rates (not shown).

Ser184 is located in the helix α9 of Bax, and is the target of several kinases, including AKT [38, 39]. The phosphorylation of Bax by AKT impairs Bax action on mitochondria, but the underlying mechanisms are not completely clear, as they depend on the presence of anti-apoptotic proteins [40, 41]. However, there is a consensus about the consequences of substituting this residue on Bax localization: Bax-S184D remains mostly cytosolic (and is rapidly degraded when expressed in yeast), while Bax-S184V (or Bax-S184A) is markedly associated with mitochondria (including in yeast).

**Figure 3:**
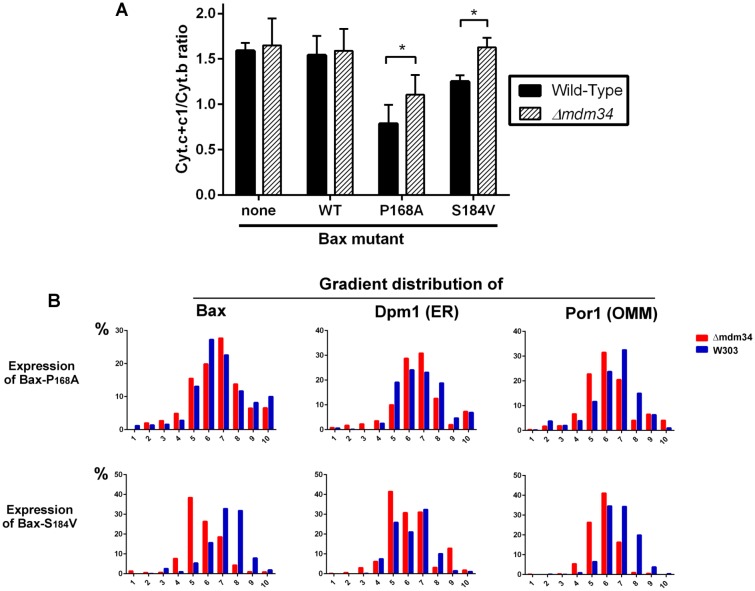
FIGURE 3: Bax-S184V looses the ability to release cytochrome c in Δ*mdm34* cells. **(A)** Cytochrome c+c1/Cytochrome b ratios measured in mitochondria from different strains. Values are averages (± s.d.) of 4 to 6 independent experiments. **(B)** Distribution of Dpm1 (ER), Por1 (OMM) and Bax on a crude mitochondrial fraction separated on sucrose density gradients. Results are representative of 3 independent experiments.

Although being largely mitochondrial, Bax-S184V was less efficient than Bax-P168A to promote the release of cytochrome c, like previously reported [40]. Furthermore, it was less efficient when expressed in the Δ*mdm34* strain than in the wild-type strain, suggesting that the Δ*mdm34* deletion had a stronger effect on Bax-S184V than on Bax-P168A (**Fig. 3A**). As negative controls, a Δ*mdm34* strain that did not express Bax, or that expressed BaxWT (which, like in non-apoptotic mammalian cells, is poorly mitochondrial), did not show any difference in residual mitochondrial cytochrome c, in comparison to wild-type.

We next analyzed the distribution of crude mitochondria preparations of strains expressing Bax-P168A and Bax-S184V on density gradients (**Fig. 3B**). Like for gradients of whole cells extracts, we observed that mitochondria-rich fractions (containing Por1), were less dense in the Δ*mdm34* strain (around fraction 6) than in the wild-type strain (around fraction 7).

The distribution of Bax-P168A was not dramatically altered by the Δ*mdm34* mutation, with a marginal inversion between the two major Bax-containing fractions (6 and 7). The Δ*mdm34* mutation caused a retention of Bax-P168A in the ER (**Fig. 2C** and **3B**), as shown by a slight increase of the co-localization of Bax and Dpm1 and a slight decrease of the co-localization of Bax and Por1 (**Table 1**). However, enough protein remained able to reach mitochondria to promote a significant release of cytochrome c, although slightly lower than in the wild-type strain (**Fig. 3A**).

**Table 1:** Co-localization of Bax with Por1 and Dpm1.

**Pearson Correlation Coefficient (p value)**	**BaxP168A v/s Por1**	**BaxP168A v/s Dpm1**
W303 (wild-type)	0.699 (<0.001)	0.428 (0.007)
Δ*mdm34*	0.592 (<0.001)	0.582 (0.001)
**Pearson Correlation Coefficient (p value)**	**BaxS184V v/s Por1**	**BaxS184 v/s Dpm1**
W303 (wild-type)	0.939 (<0.001)	0.375 (0.084)
Δ*mdm34*	0.829 (0.005)	0.845 (0.010)

The repartition of Bax, Por1 and Dpm1 on fractionated sucrose density gradients was measured in 3 independent experiments for each of the 4 strains. The co-localization is shown as the Pearson correlation coefficient between Bax and Por1 or Bax and Dpm1. Values above 0.5 indicate a good correlation. Values above 0.8 indicate a strong correlation.

The change in the distribution of Bax-S184V was more dramatic, with a marked shift towards lower density fractions (5 and 6). It is noteworthy that fraction 5, which contained 40% of total Bax-S184V present in the crude mitochondrial fraction, contained 40% of total Dpm1, but less than 30% of Por1, suggesting that a significant proportion of Bax-S184V remained localized in ER membranes associated with mitochondria. Consequently, the quantification showed a striking increase of the colocalization of Bax and Dpm1 (**Table 1**). This correlates with the total loss of capacity of Bax-S184V to release cytochrome c in the Δ*mdm34* strain (**Fig. 3A**).

Experiments reported above showed that MAMs stability affect, albeit moderately, the mitochondrial localization of Bax and its capacity to release cytochrome c. We next addressed whether cytochrome c release was, by itself, affected by MAMs stability. Our hypothesis was that, following Bax expression, the mitochondrial fraction might contain two pools of cytochrome c: unreleased cytochrome c genuinely localized in the inter-membrane space or 'incompletely released' cytochrome c that was not anymore in the inter-membrane space but not yet in the cytosol, and that was trapped in ER membranes associated with mitochondria. To discriminate between these pools, we compared the level of reduction by dithionite, a chemical reducer, with the level of reduction by NADH, a substrate of the yeast respiratory chain, that is oxidized by two inter-membrane space-facing NADH dehydrogenases, and is thus able to reduce only cytochrome c localized within the inter-membrane space [42].

**Figure 4:**
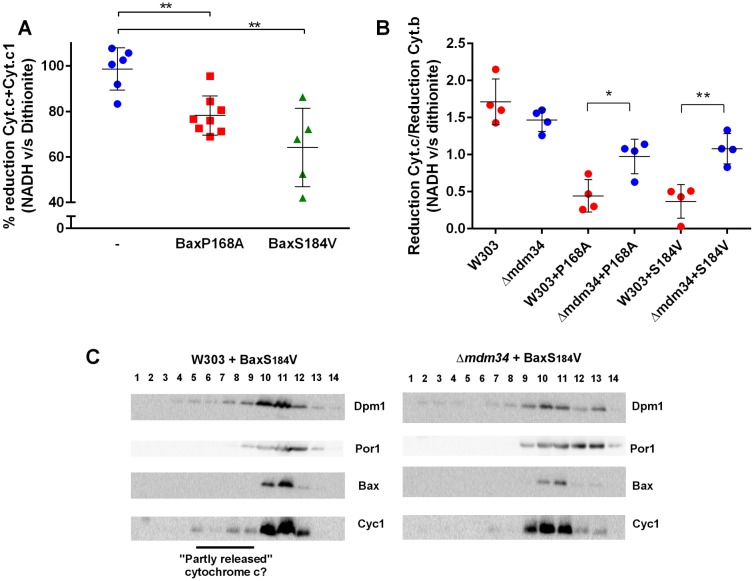
FIGURE 4: An incompletely released, MAMs-associated, pool of cytochrome c is decreased in Δ*mdm34* cells. **(A)** The fraction of NADH-reducible cytochrome c was measured by measuring reduction after adding 1 mM NADH, followed by measuring the reduction after adding dithionite on the same sample. Typically, two redox spectra with NADH and two redox spectra with dithionite were acquired sequentially, to ensure that redox ratios did not change during the time of the experiment. Each point is a different mitochondria preparation (**: significantly different p<0.01). **(B)** The ratio (reduction by NADH/reduction by dithionite) was measured for cytochrome c and for cytochrome b. The graph shows the ratio (% reduction cytochrome c) / (% reduction of cytochrome b). The values were corrected to remove the contribution of cytochrome c1, considering that the molecular ratio cyt.b/cyt.c1 is 2 and that the reduction of cytochrome c1 was proportionnal to that of cytochrome b (as they belong to the same respiratory complex). Each point is a different mitochondria preparation. (*: p<0.05; **: p<0.01). **(C)** Mitochondria were fractionated on a sucrose density gradient. A small pool of cytochrome c was found at a lower density than that of the main fraction, and may represent partly released cytochrome c, not reducible by NADH in experiments (A) and (B). The figure is representative of 3 independent experiments.

In wild-type mitochondria, as expected, 100% of cytochrome c reduced by dithionite was also reducible by NADH (**Fig. 4A**). However, in mitochondria isolated from the strain expressing Bax-P168A or Bax-S184V, a significant proportion of cytochrome c reduced by dithionite was not reduced by NADH (**Fig. 4A**). This showed that, following Bax expression, a fraction of cytochrome c, present in the crude mitochondrial fraction was not localized in the inter-membrane space, and thus is not able to receive electrons transferred from Complex III. We next tested whether there was a difference between wild-type and Δ*mdm34* strains. To attenuate the effect of variations in purity between mitochondrial preparations, we corrected the reduction of cytochrome c with the reduction of cytochrome b, an unreleased integral membrane protein that is involved in the transfer of electrons between NADH and cytochrome c (**Fig. 4B**). There was no difference between wild-type and Δ*mdm34* control strains, showing that, in the absence of Bax, all cytochrome c remained within the inter-membrane space*.* We found a different behavior in strains expressing Bax-P168A or Bax-S184V: the size of the NADH-reducible pool was decreased in the wild-type, and significantly re-increased in Δ*mdm34* (**Fig. 4B**). This showed that a pool of non NADH-reducible cytochrome c remained in the mitochondrial fraction following the expression of Bax mutants, and disappeared in the Δ*mdm34* strain: this incompletely released pool of cytochrome c was thus dependent on MAMs stability. Supporting this hypothesis, in mitochondria preparations from wild-type cells expressing Bax-S184V, but not in Δ*mdm34* expressing Bax-S184V, some cytochrome c was found in lower density fractions, that may correspond to the partly or incompletely released cytochrome c identified by spectrometry (**Fig. 4C**).

## DISCUSSION

Data reported above show that, in a model cellular system, the interaction of Bax with mitochondria is modulated by the stability of MAMs. The effects of MDM34 deletion on MAMs stability only marginally affected cell growth and viability, and did not visibly alter the mitochondrial network, contrary to the deletion of the three other ERMES components. This is in line with the proposed model for ERMES organization, where the absence of MDM34 would have less dramatic effects on the complex organization than the absence of any of the three other ones [17]. We observed that the deletion of Mdm34 was sufficient to limit the contacts between ER and mitochondria: indeed, whole cells extracts density gradients showed that the ER fraction was present at the same density as mitochondria in wild-type cells and was greatly reduced in Δ*mdm34* cells (**Fig. 1**). In spite of a moderate effect on MAMs stability, MDM34 deletion was nevertheless sufficient to alter the behavior of both Bax-P168A and Bax-S184V mutants.

Both mutants normally display a strong mitochondrial localization, but not the same level of activity (ability to release cytochrome c) that is inversely correlated to the remaining cytochrome c content of crude mitochondrial fractions. Bax-P168A is very active, as evidenced by a high ratio of cytochrome c release [28, 30, 31]. Even though its capacity to release cytochrome c is decreased in Δ*mdm34* cells, it remained high (**Fig. 3A**). On the opposite, the activity of Bax-S184V is modest [28, 40], particularly in regard to its high mitochondrial content, suggesting that its intrinsic activity is low [29, 40]. As a consequence, its capacity to release cytochrome c was almost completely abolished when expressed in Δ*mdm34* cells (**Fig. 3A**). The consequences of MDM34 deletion were thus more visible with Bax-S184V than with Bax-P168A, both in terms of Bax localization and of capacity to release cytochrome c. This revealed an unexpected aspect of Bax function: a fraction of cytochrome c that was still present in the crude mitochondrial fraction, could not be reduced by the respiratory substrate NADH. This pool of cytochrome c was detected by spectrophotometry, showing its full assembly with the haem moiety, a process taking place within mitochondria ([43] for review), and eliminating the hypothesis that it was a pool of cytochrome c not reaching mitochondria. It was therefore a pool of released cytochrome c, that remained yet associated with mitochondria. This fraction of cytochrome c likely corresponds to that detected by western-blots in lower density fractions. Interestingly, both NADH reduction and western-blots showed that the size of this fraction was markedly decreased in Δ*mdm34*, evidencing that this fraction of incompletely released cytochrome c was actually located in MAMs.

**Figure 5:**
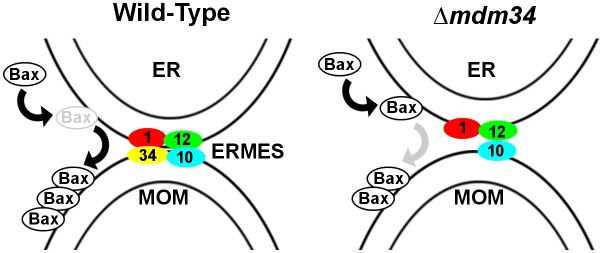
FIGURE 5: Hypothetical pathway of Bax relocation. In wild-type cells, Bax would transit through the ER before reaching the OMM through ERMES-stabilized contacts. The deletion of MDM34 would destabilize the contacts and decrease the rate of transfer to OMM, thus inducing an increase of the accumulation in the ER. We speculate that the complete loss of contacts through the deletion of other ERMES components would fully impair Bax mitochondrial relocation.

These data show that, in a model cellular system, both Bax relocation to mitochondria and cytochrome c release into the cytosol depend on the stability of MAMs, suggesting that this compartment may play a role in the regulation of Bax activity during apoptosis. Different hypotheses could explain the role of MAMs. The most direct would be that Bax passed through ER and then MAMs to reach mitochondria. The destabilization of MAMs would then decrease the transit rate, resulting in a decrease of mitochondria-located Bax and an increase of ER-located Bax (**Fig. 5**). In this hypothesis, it would be expected that the total destabilization of MAMs (through the deletion of *MDM10* or *MDM12*) would more dramatically impair Bax mitochondrial localization. Although this hypothesis is the most straightforward, other alternative hypotheses can be proposed. It has been shown that Bax could be retrotranslocated from mitochondria. Thus, the maintenance of a dynamic equilibrium of Bax localization between the cytosol, ER and mitochondria is likely. Bax retrotranslocation can be modulated through the co-expression of different variants of Bcl-xL (full-length or truncated) [29, 44]. Also, Bax phosphorylation (namely on S184 by AKT) might be a factor regulating both Bax localization [38–40] and interaction with other Bcl-2 family partners [40, 41].

These observations should now be extended to mammalian cells where it is, however, more difficult to modulate MAMs stability without affecting other processes relevant to apoptosis, such as Ca^2+^ transport [14]. Yeast could therefore establish itself as an essential complementary model for these studies.

## MATERIALS AND METHODS

The deletion cassettes of ERMES genes from the Euroscarf collection were amplified by PCR, transferred into the W303-1A strain by homologous recombination and selected for G418 resistance, and verified by PCR with primers located within the KanMX4 gene and in the 5' and 3' sequences of the ERMES gene. Like reported by others [26], the deletion of MMM1, MDM10 or MDM12 led to a rapid loss of mitochondrial DNA (*rho-/0* genotype), making these strains useless for our studies. However, we could generate stable *rho+* strains deleted for MDM34.

Wild type W303-1A (*mat*a, *ade2, his3, leu2, trp1, ura3*) and the mutant Δ*mdm34*::kanMX4 were transformed with plasmids pYES3-BaxWT, pYES3-BaxP168A, or pYES3-BaxS184V [27,28]. Co-transformations with the plasmid pYES2-Bcl-xL were also done.

Cells were pre-grown in YNB medium (Yeast Nitrogen Base 0.17%, ammonium sulfate 0.5%, potassium dihydrogenphosphate 0.1%, Drop-Mix 0.2%, auxotrophic requirements 0.01%, pH 5.5, supplemented with 2% glucose), and then transferred in the same YNB medium supplemented with 2% lactate, instead of glucose, to obtain an optimal differentiation of mitochondria. When the cultures reached the mid-exponential growth phase (O.D. at 550 nm between 4 and 6), they were diluted in the same medium down to 0.7 - 0.8 O.D. and added with 0.8% galactose, to induce Bax expression.

Whole lysates were prepared from 10-20 mL cultures. Bax expression was induced for 5 hours. Cells were harvested and washed in the RB buffer (0.6 M mannitol, 2 mM EGTA, 10 mM tris-maleate 10 mM, pH 6.7, anti-protease cocktail (Complete-Mini, Roche)), re-suspended in 0.5 mL of ice-cold RB buffer and added with 0.40 mm-mesh HCl-washed glass beads (2/3 of the total volume). Cells were broken in a Tissue Lyser (30 Hz, 3 minutes). The homogenate was centrifuged at 500 × g (10 minutes) to eliminate residual beads, unbroken cells and nuclei. Protein concentration was measured by the Lowry method.

Crude mitochondria fractions were isolated from 2L-cultures. Bax was expressed for 14 hours (see [28] for a detailed protocol). Briefly, cells were converted to spheroplasts and homogenized. Two series of low-speed/high-speed centrifugations allowed to obtain a crude mitochondria pellet.

1-3 mg of proteins from cell lysates or isolated mitochondria (10 mg/mL) were layered on the top of a 10 mL-density gradient in Beckmann SW41 tubes. Both sucrose and Optiprep (Stemcell Technologies) gradients were done (see results). Gradients were centrifuged overnight at 135,000 × g (28,000 rpm). Fractions were recovered with an Auto-densiflow II collector (Buchler), precipitated with 0.3 M TCA, washed with 100µL cold acetone and solubilized in Laemmli buffer.

Cytochromes contents of isolated mitochondria were measured by differential redox spectrophotometry in a double-beam spectrophotometer Varian Cary 4000 and graphically corrected, as described previously [29].

For western-blots, proteins were separated on 12.5% SDS-PAGE, transferred onto nitrocellulose, saturated in PBST/milk or TBST/BSA (depending on antibodies) and blotted with the following antibodies: anti-human Bax 2D2 (Santa-Cruz, 1/5,000 dilution), anti-yeast porin (Por1) (Novex, 1/50,000 dilution), anti-yeast Phosphoglycerate Kinase (Pgk1) (Novex, 1/10,000 dilution), anti-yeast Dolichol Phosphate Mannose Synthase (Dpm1) (Novex, 1/5,000 dilution), anti-yeast Cytochrome c (Cyc1) (Custom made, Millegen, 1/5,000 dilution), anti-yeast Cytochrome c Oxidase subunit II (Cox2) (Invitrogen, 1/5,000 dilution), anti-yeast carboxypeptidase Y (Cpy1) (Invitrogen, 1/10,000 dilution). Horse radish peroxidase-coupled secondary antibodies (Jackson Laboratories, 1/10,000 dilution) were revealed by ECL (Luminata Forte, Millipore), visualized with a digital camera (G-Box, Syngene), and quantified using Image J software. Statistical analyzes and figures were made with GraphPad Prism 6 software.

## SUPPLEMENTAL MATERIAL

Click here for supplemental data file.

All supplemental data for this article are available online at http://www.microbialcell.com/researcharticles/2019a-legiot-microbial-cell/.

## Abbreviations:

ER: – endoplasmic reticulum,
ERMES: – ER-mitochondria encounter structure,
MAM: – Mitochondria-Associated Membrane,
OMM: – outer mitochondrial membrane.
