# Supplementary material for: Mitochondria-Associated Membranes (MAMs) are involved in Bax mitochondrial localization and cytochrome c release
Source: Microb Cell. 2019 Mar 15;6(5):257–66. doi: 10.15698/mic2019.05.678 (PMC6506693; doi:10.15698/mic2019.05.678)

Supplementary Figure S1: Mitochondrial network morphology in *Δmdm34* cells.
Cells expressing a mitochondria-addressed Idh1-GFP fusion were observed under an epifluorescence microscope.

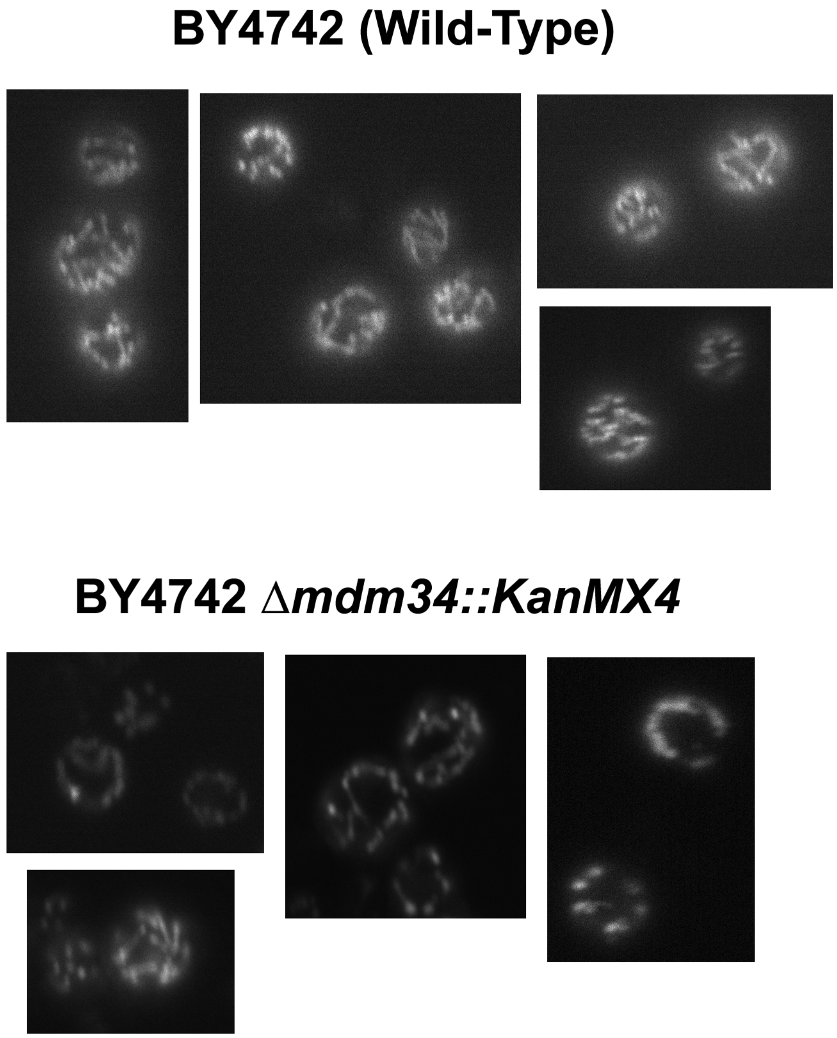

Supplement: Supplementary file 1 [file mic-06-257-s01.pdf]
